# Carotid Artery Injury With a Migrating Venous Missile Embolus: A Unique Sequela of Shotgun Neck Trauma

**DOI:** 10.7759/cureus.114129

**Published:** 2026-08-07

**Authors:** Jamie B Hadley, Robert S Martin

**Affiliations:** 1 Acute Care Surgery, Atrium Health Wake Forest Baptist, Winston-Salem, USA

**Keywords:** bullet embolism, carotid artery injury, foreign body in pulmonary artery, penetrating neck trauma, shotgun injury

## Abstract

Ballistic injuries to the neck carry a significant risk of injury to the airway, vascular, and aerodigestive structures. We report the case of a young adult who sustained a close-range shotgun injury to the left anterior neck. Despite hemodynamic stability and an initially intact airway, CT angiography (CTA) was unable to adequately visualize the carotid artery or internal jugular vein (IJV) because of extensive metallic artifact from multiple retained birdshot pellets. The patient underwent urgent operative exploration, during which numerous pellets were identified within the soft tissues, including several that were adherent to the left IJV and common carotid artery. Multiple venous injuries were repaired, and a full-thickness carotid artery injury required segmental resection with primary end-to-end anastomosis. Postoperatively, a radiographic fragment near the cardiac border was confirmed by echocardiography to be a venous missile embolus lodged in the right ventricular apex. After multidisciplinary review, the fragment was managed nonoperatively. The patient recovered without neurologic deficits and was discharged home the following day. This case emphasizes the importance of operative exploration when CTA is limited by artifact and highlights evidence-based selective nonoperative management of intracardiac and intrapulmonary missile emboli.

## Introduction

Penetrating neck trauma presents a unique diagnostic challenge because of the high density of vital vascular, aerodigestive, and neurologic structures within a relatively confined anatomic space. Although CT angiography (CTA) has become central to the selective evaluation of hemodynamically stable patients without hard signs of injury, operative exploration remains necessary when imaging is inconclusive, and injury to critical structures cannot be reliably excluded [1-3]. In such cases, the inability to reliably exclude injury may necessitate operative exploration despite an otherwise reassuring clinical examination.

Intravascular migration of ballistic fragments represents a rare and unpredictable complication of penetrating trauma. Missile emboli may enter the venous or arterial circulation and migrate to the heart, pulmonary vasculature, or systemic circulation, with clinical presentations ranging from incidental radiographic findings to life-threatening complications [4-6]. Because of their rarity, management is largely guided by case reports and small case series, and the optimal treatment of asymptomatic intracardiac and pulmonary missile emboli remains uncertain [4,7].

We present the case of a close-range shotgun injury to the neck complicated by significant carotid artery and internal jugular venous injuries that could not be adequately evaluated by CTA because of extensive metallic artifact, as well as simultaneous ballistic embolization to the right ventricle and pulmonary vasculature. This case highlights the limitations of cross-sectional imaging in penetrating injuries involving multiple ballistic fragments, the importance of operative exploration when critical vascular structures cannot be reliably assessed, and the role of selective nonoperative management of asymptomatic missile emboli.

## Case presentation

This is the case of a patient in their 20s who presented to our urban Level I trauma center in Winston-Salem, North Carolina, as a transfer from an outside hospital (OSH) following a close-range shotgun injury to the left anterior neck. On presentation to the OSH, they were hemodynamically stable with a Glasgow Coma Scale (GCS) score of 15, and airway, breathing, and circulation were intact. Emergency department documentation noted mild hoarseness of their voice. The patient was intubated before transfer to ensure airway protection given the proximity of the injury to critical aerodigestive structures.

**Figure 1:**
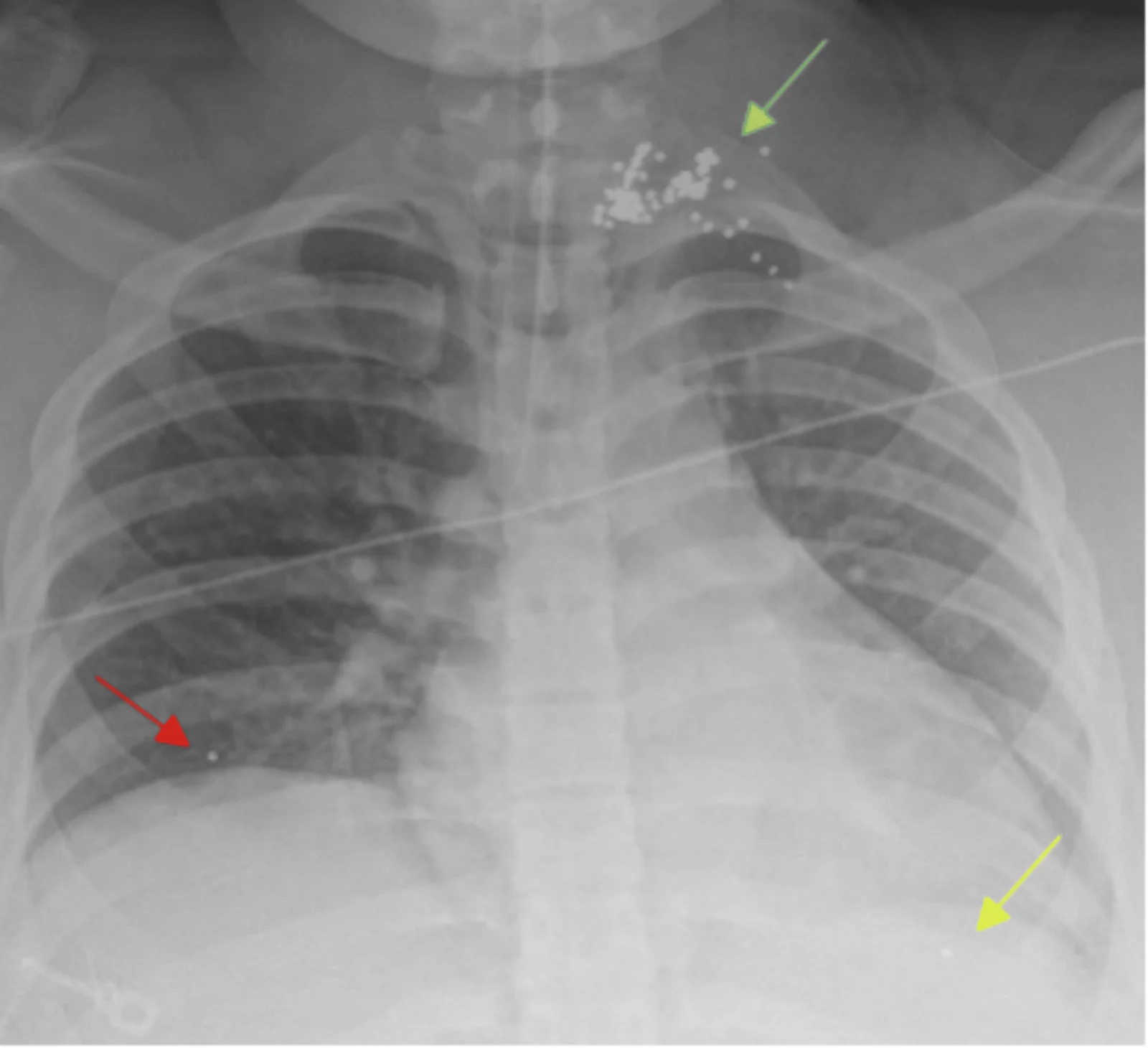
Chest X-ray in the trauma bay showing birdshot in the neck (green arrow), apex of the heart (yellow arrow), and right lung periphery (red arrow).

On arrival at our institution, they remained hemodynamically stable with bilateral breath sounds and a protected airway, with an endotracheal tube (ETT) supporting oxygenation and ventilation. There was a 2.5-cm missile wound to the left neck, just lateral to the midline, without active bleeding, an expanding hematoma, air bubbling, or salivary drainage. Primary and secondary surveys were unremarkable aside from the neck wound. Initial plain-film imaging revealed multiple small pellets in the left neck and two additional metallic fragments: one in the periphery of the right lung and one near the left chest (Figure 1).

**Figure 2:**
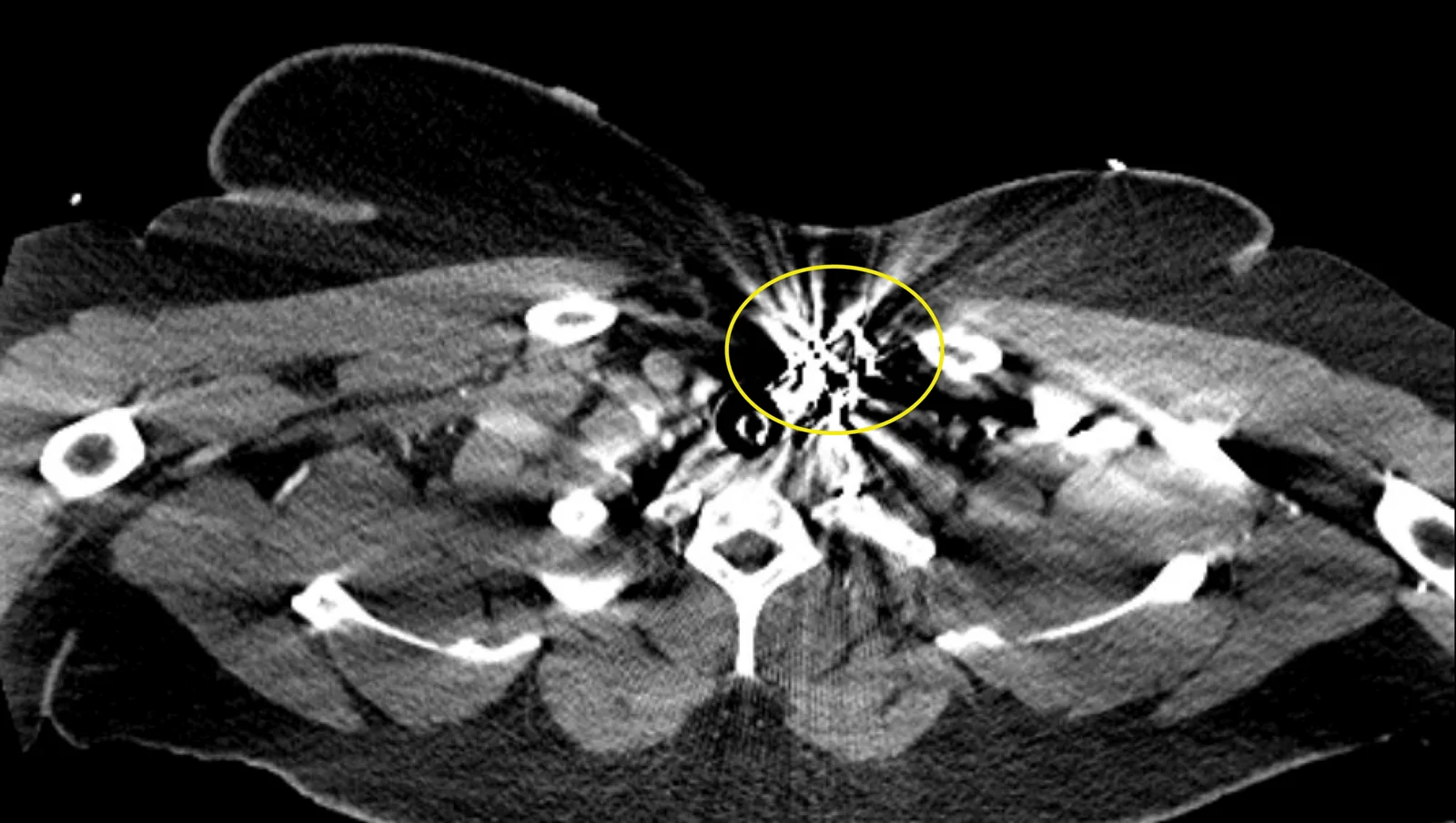
Representative CT image showing birdshot causing artifact over the carotid artery and jugular vein, obscuring visualization of vessel integrity.

**Figure 3:**
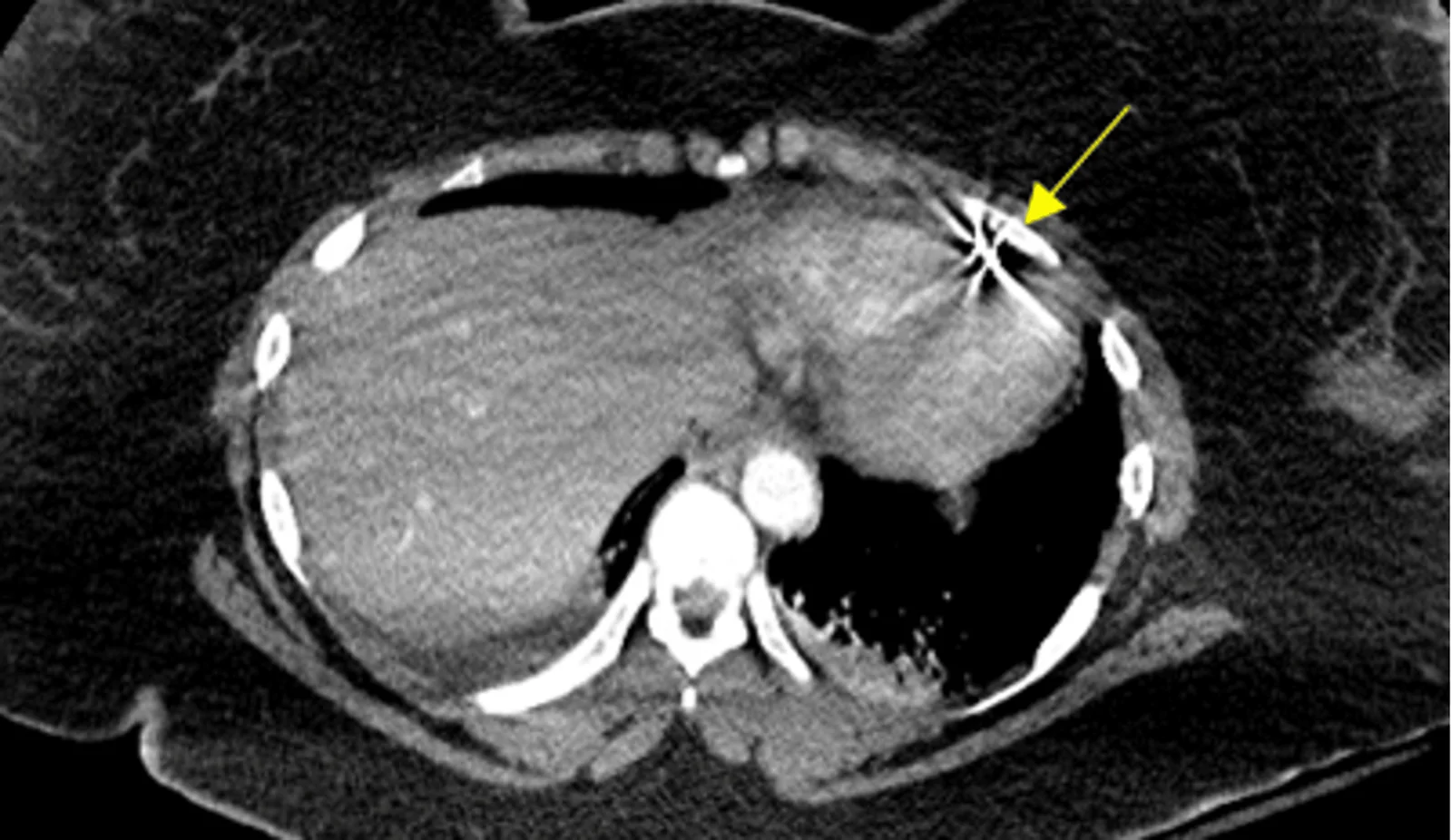
Representative CT image of a pellet at the apex of the heart.

**Figure 4:**
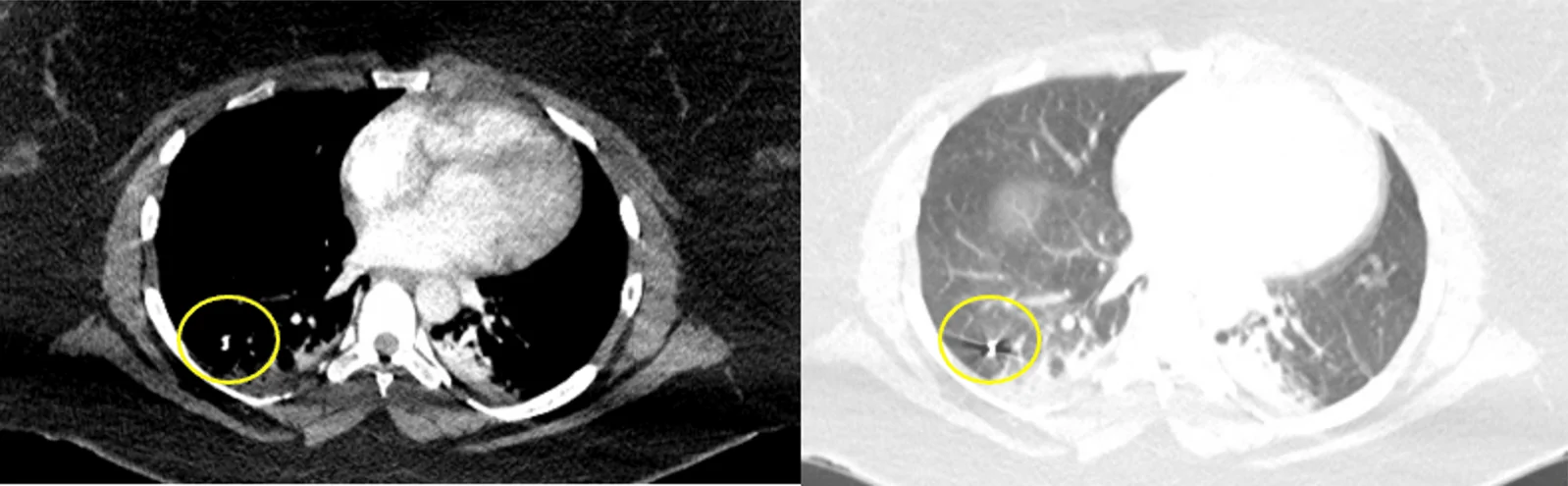
CT images in soft tissue and lung windows demonstrating a pellet in the right lung periphery. Left image: soft tissue window. Right image: lung window.

Given the absence of hard signs of vascular or aerodigestive injury, CTA of the head, neck, and chest was performed (Figure 2, Figure 3, Figure 4). CT was limited by metallic artifact from multiple pellets, preventing adequate visualization of the carotid artery and internal jugular vein (IJV). Pneumomediastinum was present, tracking from the neck wound into the superior mediastinum. Because vascular integrity could not be reliably assessed, the patient was taken urgently to the operating room for left neck exploration. Dissection of the left neck revealed numerous metallic pellets embedded throughout the soft tissues. Several pellets were adherent to the anterior wall of the left IJV and left common carotid artery.

Control of the jugular vein was obtained first. Removal of embedded pellets exposed multiple small venous injuries, each of which was repaired with interrupted 6-0 Prolene sutures. The vein was patent after repair. Attention was then directed to the carotid artery. After proximal and distal vascular control had been achieved, the patient received 5,000 units of intravenous heparin, and the carotid artery was clamped. Exploration revealed a full-thickness anterior wall injury requiring circumferential resection of approximately 1 cm of the vessel. Minimal thrombus was present and was removed. The lumen was flushed and back-bled to ensure patency. A tension-free primary end-to-end anastomosis was performed using running 6-0 Prolene.

Completion of the neck exploration demonstrated no injury to the trachea or cervical esophagus. The wound was closed in multiple layers, and the skin was closed with staples. The patient was transported intubated to the trauma ICU, and ventilatory support was successfully weaned overnight. They passed a spontaneous breathing trial the following morning and were extubated to a nasal cannula. Following extubation, the patient was alert with a GCS score of 15 and exhibited no neurologic deficits.

**Figure 5:**
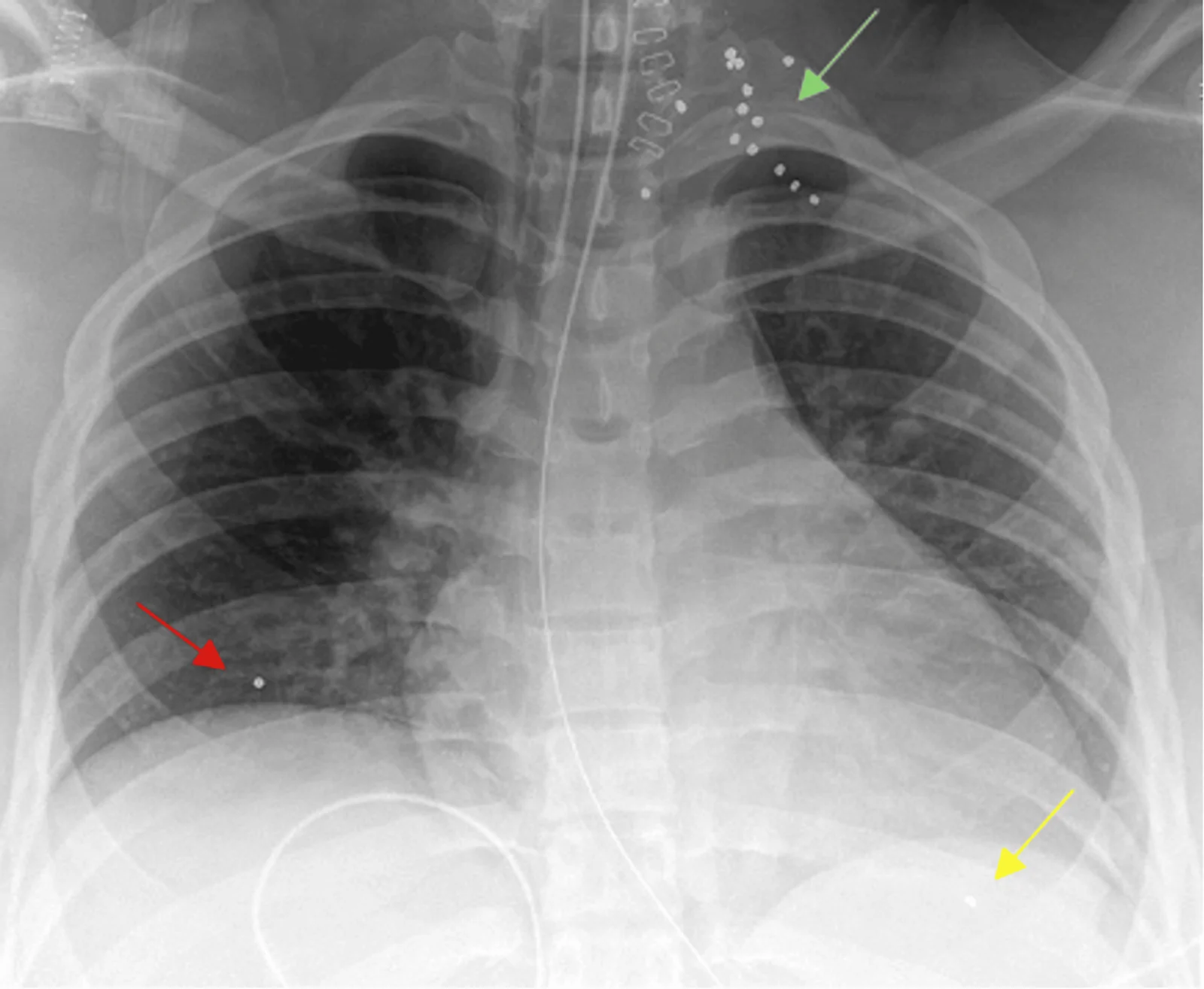
Postoperative chest X-ray showing stable locations of the pellets within the neck (green arrow), apex of the heart (yellow arrow), and right lung periphery (red arrow).

A postoperative chest radiograph was obtained to reevaluate the metallic fragments in the right lateral chest and mediastinum (Figure 5). A metallic fragment remained near the cardiac silhouette and was thought to represent a pellet embolus originating from the IJV. The fragment at the periphery of the right lung was also believed to represent an embolus originating from the IJV injury. Both fragments remained unchanged in position compared with the preoperative imaging. Echocardiography confirmed the fragment’s location at the right ventricular apex, and cardiothoracic surgery (CTS) was consulted to determine whether intervention was required. After multidisciplinary discussions involving CTS and interventional cardiology, the decision was made to manage the intracardiac missile nonoperatively, as the risk of retrieval outweighed the potential consequences of embolization to the pulmonary vasculature. The patient recovered uneventfully and was discharged home on postoperative day 2. At the one-week follow-up visit, the patient was doing well, and the staples were removed.

## Discussion

Shotgun pellet (birdshot) embolism to the pulmonary vasculature is a rare complication of penetrating trauma, most often occurring via venous entry after a gunshot wound, and may present with a spectrum of symptoms ranging from asymptomatic to those of classic pulmonary embolism. The literature describes both incidental findings and cases with respiratory distress, chest pain, or hemodynamic instability, but many patients remain asymptomatic, especially if the embolus is small or peripheral, as in this case [4,5]. The diagnosis relies on a high index of suspicion, especially when there is an odd number of external missile wounds or when imaging fails to localize the missile at the expected site. Chest radiography, CTA, and echocardiography are critical for identifying and localizing the embolus, with arteriography sometimes used for confirmation. Migratory or occult emboli can complicate diagnosis, as highlighted by cases of missile migration within the pulmonary circulation [5-8].

Management strategies for missile emboli depend on symptoms, hemodynamic status, and the risk of complications. Intervention is indicated for symptomatic patients, those with hemodynamic compromise, or those at risk of infarction, infection, or other sequelae [7,9-11]. For asymptomatic and hemodynamically stable patients, such as our patient, observation is reasonable and has been associated with good outcomes. Several case series and reviews have reported no adverse sequelae in observed patients [4,7,12]. Surgical embolectomy (open or endovascular) is preferred for symptomatic or high-risk cases, and endovascular retrieval is increasingly used [5,6,8,13]. Clinical outcomes are generally favorable, and most patients undergoing intervention (surgical or endovascular) recover well, with low mortality and minimal long-term sequelae [5,7,13]. Asymptomatic patients managed conservatively also tend to do well, with no reported adverse outcomes during follow-up [7,12].

In our case, the right lung missile was located peripherally, and there was no evidence of adjacent infarction. One might assume similar behavior from the cardiac missile, as we presume the lung missile had traveled from the IJV to the heart before lodging in the pulmonary vasculature. Despite our attempts to bring the patient back for follow-up plain-film imaging to assess pellet stability, we were unable to schedule a follow-up appointment because of patient-related factors. This challenge reflects the realities of our trauma population, in which unstable housing, limited transportation, competing social priorities, and inconsistent access to primary care frequently impede reliable outpatient follow-up. As a result, longitudinal surveillance after injury can be difficult despite clear discharge instructions and coordinated outreach efforts.

There is a lack of large-scale randomized data specific to shotgun pellet embolism to the pulmonary vasculature, and management is guided by case reports, small case series, and extrapolation from general pulmonary embolism guidelines. Further research is needed to clarify optimal management and long-term outcomes [4,9].

This case report has several important limitations inherent to single-patient descriptions of rare traumatic presentations. First, the patient’s close-range shotgun injury resulted in extensive metallic artifact, significantly limiting the diagnostic utility of CTA. Although this limitation is itself clinically relevant, it restricts our ability to comment on imaging findings that might otherwise be generalizable to similar injuries. Second, the decision to proceed with operative exploration was influenced by local resources, clinical experience, and institutional practice patterns; therefore, operative thresholds and management strategies may vary across trauma centers. Third, although the patient sustained a venous missile embolus to the right ventricle, the fragment remained stable and required no intervention. As a result, our case cannot address the broader spectrum of potential embolization complications or procedural techniques for retrieval. Fourth, long-term follow-up was limited to a short-interval postoperative evaluation, preventing assessment of delayed complications such as pseudoaneurysm formation, carotid stenosis, late neurologic events, or fragment migration. Finally, the nature of shotgun injuries, characterized by highly variable patterns of tissue penetration, pellet dispersion, and energy transfer, limits the generalizability of this case to other ballistic mechanisms.

## Conclusions

This case highlights the diagnostic and therapeutic challenges of close-range shotgun injuries to the neck, particularly when metallic artifact limits the accuracy of cross-sectional imaging. Despite hemodynamic stability and the absence of hard signs of vascular injury, the inability to adequately evaluate the carotid artery and IJV on CTA necessitated operative exploration, during which multiple vascular injuries were identified and repaired. The concomitant finding of a venous missile embolus lodged in the right ventricle, as well as within the pulmonary vasculature, further underscores the unpredictable trajectory of ballistic fragments and the importance of maintaining a high index of suspicion for intravascular migration. Successful carotid reconstruction and favorable neurologic recovery in this patient demonstrate the effectiveness of prompt surgical intervention when injury to critical structures cannot be reliably excluded. This case reinforces the need for careful clinical assessment, selective operative management, and multidisciplinary collaboration in the evaluation and treatment of penetrating neck trauma complicated by ballistic embolization.
